# Efficacy and safety of extracorporeal membrane oxygenation for burn patients: a comprehensive systematic review and meta-analysis

**DOI:** 10.1093/burnst/tkac056

**Published:** 2023-03-01

**Authors:** Xue Heng, Peng Cai, Zhiqiang Yuan, Yizhi Peng, Gaoxing Luo, Haisheng Li

**Affiliations:** Institute of Burn Research, State Key Laboratory of Trauma, Burns and Combined Injury, Southwest Hospital, Third Military Medical University (Army Medical University), Chongqing, 400038, China; Department of Intensive Care Medicine, PLA 80th Group Army Hospital, Wei Fang City, Shan Dong Province, 261000, China; Institute of Burn Research, State Key Laboratory of Trauma, Burns and Combined Injury, Southwest Hospital, Third Military Medical University (Army Medical University), Chongqing, 400038, China; Institute of Burn Research, State Key Laboratory of Trauma, Burns and Combined Injury, Southwest Hospital, Third Military Medical University (Army Medical University), Chongqing, 400038, China; Institute of Burn Research, State Key Laboratory of Trauma, Burns and Combined Injury, Southwest Hospital, Third Military Medical University (Army Medical University), Chongqing, 400038, China; Institute of Burn Research, State Key Laboratory of Trauma, Burns and Combined Injury, Southwest Hospital, Third Military Medical University (Army Medical University), Chongqing, 400038, China

**Keywords:** Extracorporeal membrane oxygenation, Burns, Acute respiratory distress syndrome, Inhalation injury

## Abstract

**Background:**

Respiratory and circulatory dysfunction are common complications and the leading causes of death among burn patients, especially in severe burns and inhalation injury. Recently, extracorporeal membrane oxygenation (ECMO) has been increasingly applied in burn patients. However, current clinical evidence is weak and conflicting. This study aimed to comprehensively evaluate the efficacy and safety of ECMO in burn patients.

**Methods:**

A comprehensive search of PubMed, Web of Science and Embase from inception to 18 March 2022 was performed to identify clinical studies on ECMO in burn patients. The main outcome was in-hospital mortality. Secondary outcomes included successful weaning from ECMO and complications associated with ECMO. Meta-analysis, meta-regression and subgroup analyses were conducted to pool the clinical efficacy and identify influencing factors.

**Results:**

Fifteen retrospective studies with 318 patients were finally included, without any control groups. The commonest indication for ECMO was severe acute respiratory distress syndrome (42.1%). Veno–venous ECMO was the commonest mode (75.29%). Pooled in-hospital mortality was 49% [95% confidence interval (CI) 41–58%] in the total population, 55% in adults and 35% in pediatrics. Meta-regression and subgroup analysis found that mortality significantly increased with inhalation injury but decreased with ECMO duration. For studies with percentage inhalation injury ≥50%, pooled mortality (55%, 95% CI 40–70%) was higher than in studies with percentage inhalation injury <50% (32%, 95% CI 18–46%). For studies with ECMO duration ≥10 days, pooled mortality (31%, 95% CI 20–43%) was lower than in studies with ECMO duration <10 days (61%, 95% CI 46–76%). In minor and major burns, pooled mortality was lower than in severe burns. Pooled percentage of successful weaning from ECMO was 65% (95% CI 46–84%) and inversely correlated with burn area. The overall rate of ECMO-related complications was 67.46%, and infection (30.77%) and bleedings (23.08%) were the two most common complications. About 49.26% of patients required continuous renal replacement therapy.

**Conclusions:**

ECMO seems to be an appropriate rescue therapy for burn patients despite the relatively high mortality and complication rate. Inhalation injury, burn area and ECMO duration are the main factors influencing clinical outcomes.

Highlights

- ECMO seems to be an appropriate rescue therapy for burn patients.
- The mortality of ECMO was 49% in burn populations and was related to inhalation injury and ECMO duration.
- Pooled percentage of successful weaning from ECMO was 65% and inversely correlated with burn area.
- The rate of ECMO-related complications was 67.46%, and infection and bleeding were the two most common complications.

## Background

Burns are a major cause of injuries worldwide. It is estimated that >300,000 people die from burns every year worldwide [1]. The overall mortality of admitted burn patients is 3–8% [2], which increases to 30% in burns with inhalation injury [3] and about 40–70% in severe burns [3,4]. Multiple organ failure, mainly lung, heart and kidney, are the main complications and causes of death in burn patients. A survey of 6212 burn deaths showed that burn shock, cardiogenic shock or lung injury, and sepsis or multiple organ failure were the main causes of death within the first week, weeks 1 to 2, and after 2 weeks, respectively [2]. The incidence of burn-induced acute respiratory distress syndrome (ARDS) is reported to be 2–53% and mortality is 14–68% [5]. Cardiac stress, characterized with tachycardia, systolic dysfunction, increased cardiac output and energy expenditure, is the hallmark of severe burns and persists for up to 3 years after burns [6]. Cardiac dysfunction is associated with poor outcomes in burn populations [7]. The pathophysiology of respiratory and circulatory dysfunction is manifold. Severe burns can lead to hypermetabolic stress, increased inflammatory response, elevated stress hormones (vasoconstrictors, glucocorticoid) and increased microvascular permeability [8]. The subsequent massive fluid resuscitation and substantial leakage lead to serious tissue edema and hemodynamic instability [7]. This can further cause poor perfusion and ischemia of vital organs. Furthermore, inhalation injury and electrical burns can directly cause injury to lungs, blood vessels and heart. Severe infection and sepsis can potentially cause ARDS and circulatory dysfunction in the late phase of burns [9]. Therefore, effective respiratory and circulatory supports are crucial to the survival of severe burns.

Extracorporeal membrane oxygenation (ECMO) is an alternative therapy for respiratory and circulatory supports for cases where conventional treatments have failed. ECMO can be used to drain venous blood from the body to an oxygenator device through a central venous catheter, re-oxygenate and remove excess CO_2_, and pump the blood back into body through another central venous catheter (veno–venous ECMO, VV ECMO) or an arterial catheter (veno–arterial ECMO, VA ECMO). Therefore, VV ECMO mainly plays the role of ‘artificial lung’ and VA ECMO can simultaneously function as ‘artificial heart and lung’. During ECMO, the parameter of mechanical ventilation is set as very low tidal volume and respiratory frequency, minimizing ventilator-induced lung injury and providing a chance of rest and recovery for the lung [10]. Recent systematic reviews showed that ECMO could significantly reduce the in-hospital mortality of severe ARDS in adults [11,12]. However, there is not sufficient strong evidence of recommendation for or against ECMO in different guidelines [13–15].

Although ECMO was first used in burn patients in 1976 [16], all the studies on ECMO in burn populations were retrospective and had a low sample size. Previously, a narrow systematic review preliminarily found that burn patients with inhalation injuries and revised Baux scores >90 may benefit from ECMO [17]. However, they only focused on the clinical outcome of mortality, only included studies with revised Baux scores and had several case series with possible publication bias and duplicated studies. The efficacy and safety of ECMO in total burn populations still need further investigation. A recent survey in North American burn centers found that the major barrier to ECMO application in burn patients was still the inadequate and weak clinical evidence [18]. In this study, we performed a comprehensive systematic review and meta-analysis of published studies with a larger sample size and low publication bias. Our aim was to clarify: (1) if ECMO could improve clinical outcomes, including in-hospital mortality and rate of successful weaning off and (2) the safety of ECMO in burn patients, mainly indicated by common complications.

## Methods

### Search strategy

This study was conducted according to Preferred Reporting Items for Systematic Reviews and Meta-Analyses (PRISMA) guidelines [19]. The review protocol was prospectively registered with PROSPERO (CRD42022313679). A comprehensive literature search was performed in PubMed, Web of Science and Embase from inception to 18 March 2022, using search strategies as follows: ‘burns’, ‘burn’, ‘inhalation injury’ in Title/Abstract AND ‘extracorporeal membrane oxygenation’, ‘ECMO’, ‘extracorporeal life support’, ‘ECLS’ in Title/Abstract. The detailed search strategies of every database can be found in supplementary Table S1, see online supplementary material. Furthermore, the citation lists of all relevant studies were evaluated to screen for any additional work that should be included.

### Eligibility criteria

Articles were included if they studied burn patients with or without inhalation injury undergoing ECMO, and clearly reported mortality. Exclusion criteria were as follows: case report, case series with <5 patients, not in English or Chinese, surveys, conference abstracts, review articles and studies without full text. As studies conducted by the same institution in different years may have overlapping patients, in such cases, only the newest study with the longest study period and the largest sample size was included and the others were excluded. Case series (< 5 patients) were excluded because this type of study may have a strong publication and selection bias. The articles were independently reviewed by two authors (CP and HX). Whenever disagreements over inclusion of a study arose, they were resolved through discussion or the involvement of a third author (YZQ).

### Data collection

In this study, two authors (HX and CP) independently collected data, and conflicts between the two authors’ data were resolved by consensus or by a third author (LHS). The following data were extracted from articles and pooled: study features (first author, publication year, country or region of ECMO center, study type, study period), patient demographics (sample size, proportion of males, mean/median age, percentage inhalation injury, burn area), ECMO information [indication, mode, starting time post injury, duration, simultaneous application of continuous renal replacement therapy (CRRT)] and outcomes (mortality, successful weaning off, complications). Additional data were requested from corresponding authors if necessary.

### Outcomes

In-hospital mortality was the primary outcome. Successful weaning from ECMO and complications associated with ECMO were secondary outcomes.

### Assessment of risk of bias and certainty of evidence

A prevalence systematic review checklist developed by the Joanna Briggs Institute (JBI) [20] was used to assess the articles’ quality independently by two investigators (HX and CP). If the JBI score was <7, the study was also excluded from the meta-analysis. In order to assess publication bias, funnel plots were used in conjunction with Egger’s test. Forest plots, Chi-squared test and *I^2^* statistics were used to identify statistical heterogeneity. According to the *I^2^* statistics, there was low heterogeneity for ≤ 40%, moderate heterogeneity for 40–70% and considerable heterogeneity for ≥70%. Evidence certainty was assessed by the GRADE (Grading of Recommendations, Assessments, Developments and Evaluations) approach.

### Data analysis

Statistical analyses were performed by R Studio software (Version 3.6.1, R Studio, Inc. Boston) using the packages *meta* (v4.12–0) and *metafor* (v0.0.9000). Meta-analyses were conducted using inverse-variance weighted random-effects models. Clopper–Pearson methods were used to calculate 95% confidence intervals (CIs). Outcomes of mortality and successful weaning off ECMO are presented as pooled proportions with 95% CIs. A leave-one-out sensitivity analysis was conducted to determine the impact of individual studies on the overall effect, iteratively removing one study at a time. A planned subgroup meta-analysis was performed to pool the mortality and rate of successful weaning off in different groups of burn patients and explore potential sources of heterogeneity. For mortality, we assessed the following subgroups stratified by age (pediatric and adult), burn severity (minor burns: adult total body surface area (TBSA) <30%, pediatric TBSA <15%; moderate burns: adult TBSA 30–50%, pediatric TBSA 15–30%; severe burns: adult TBSA >50%, pediatric TBSA >30%), inhalation injury (percentage of inhalation injury ≥50%, percentage of inhalation injury <50%) and ECMO duration (≥10 days and <10 days). For the rate of successful weaning off, only the subgroup with burn severity was analyzed. Meta-regression was also conducted to detect where the potential factor for heterogeneity originated. The statistical significance was set as 0.05.

## Results

### Study details and demographics

**Figure 1:**
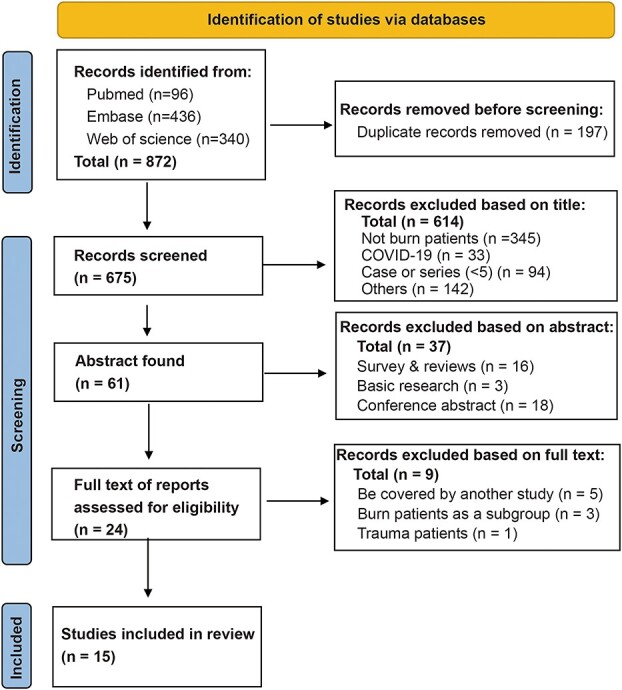
Flow diagram of study selection

A total of 872 citations were obtained and 675 documents were further evaluated after deduplication. After further step-by-step screening by title, abstract and full text, 15 articles fulfilled inclusion criteria and detailed data were extracted for systematic review **(**Figure 1). Five studies [21–25] were excluded because of overlapping patients to other studies [26–28]. All 15 articles were retrospective observational studies **(**Table 1). Most studies (12/15) were published after 2015 and were single-center studies.

The characteristics of the 318 patients included are outlined in Table 1. Gender distribution was reported in 12 studies [27,29–39] and the pooled percentage of males was 74.18% (135/182). Mean/median age ranged from 21.8 to 51 years in adults from 10 studies [29,30,32–39] and from 2.53 to 5.9 years in pediatrics from 3 studies [27,31,40]. Mean/median burn area ranged from 17% to 89% TBSA in adults from 9 studies [29,30,32–37,39] and from 17% to 50.2% TBSA in pediatrics from 3 studies [27,31,40]. Inhalation injury accounted for 49.61% (64/129) in burn patients from 12 studies [27,29–37,39,40].

### ECMO features

As shown in Table 2, severe ARDS, defined as arterial oxygen partial pressure/fraction of inspired oxygen (PaO_2_/FiO_2_) < 100 mmHg, was the most common indication of ECMO. Others included severe hypoxemia, cardiac shock and lung infection. PaO_2_/FiO_2_ before ECMO ranged from 59 to 111.8 mmHg in 9 studies [27,29,31–33,35,37,39]. Seven studies reported the median/mean initiation time as 26.5 h to 10.2 days after injury [27,29,31–33,35,37] and two studies reported it as 4 to 5 days after admission [38,39]. The median/mean duration of ECMO varied between 90 h and 18 days in adults from nine studies [29,30,32,33,35–39] and between 165.2 h and 11 days in pediatrics from 3 studies [27,31,40]. ECMO mode was reported in 170 patients from 11 articles [29–39]. VV ECMO was the most common mode (75.29%, 128/170), followed by VA ECMO (21.76%, 37/170).

**Table 1:** Patient characteristics of included studies

**No.**	**Population**	**First author (year)**		**Study period**	**Country/regions**	**Study center**	**Study design**	**Study population**	**Sample size (*n*)**	**Gender (male, *n*)**	**Median age (years)**	**Median burn area (%TBSA)**	**Inhalation injury (*n*)**
1	Adult	Li (2021) [29]		2014–2020	China mainland	Southwest hospital	Retrospective	Burns	5	5	48.2	58.8	4
2	Adult	Szentgyorgyi (2018) [33]		2011–2017	UK	Wythenshawe Hospital	Retrospective	Burns	5	3	32.4	27.8	5
3	Adult	Dadras (2019) [32]		2017–2019	German	BG University Hospital Bergmannsheil	Retrospective	Burns	8	6	48	37	7
4	Adult	Ainsworth (2018) [36]		2012–2017	USA	US Army Institute of Surgical Research	Retrospective	Burns	14	10	36	27	4
5	Adult	Hsu (2017) [37]		NR	Taiwan	Tri-Service General Hospital	Retrospective	Stun grenade explosion	6	5	43.3	89	5
6	Adult	Chiu (2017) [35]		2015	Taiwan	Taipei Veterans General Hospital	Retrospective	Formosa Water Park dust explosion	5	4	21.8	82.9	5
7	Adult	Soussi (2016) [39]		2013–2016	France	Hôpital Saint-Louis	Retrospective	Burns	11	10	51	31	6
8	Adult	Marcus (2019) [30]		2012–2018	USA	Brooke Army Medical Center	Retrospective	Burns	20	14	32	30	2
9	Adult	Nehra (2009) [28]		1990–2008	USA	Massachusetts General Hospital	Retrospective	Burns and trauma	10	NR	NR	NR	NR
10	Adult	Burke (2017) [38]		1999–2015	ELSO	ELSO	Retrospective	Burns	58	43	37	NR	NR
11	Adult	Nosanov (2017) [34]		2002–2011	USA	National burn registry of America	Retrospective	Burns	30	24	38.9	17	16
12	Pediatrics	Eldredge (2019) [31]		2006–2016	USA	A regional Burn Trauma Intensive Care Unit	Retrospective	Burns	8	6	5.9	17	3
13	Pediatrics	Kane (1999) [27]		1991–1996	USA	Shriners burns institution, Cincinnati Unit	Retrospective	Burns	12	5	2.53	50.2	4
14	Pediatrics	Pierre(1998) [40]		1992–1995	USA	Shriners burns institution, Galveston Unit	Retrospective	Burns	5	NR	4.3	41.8	3
15	Pediatrics	Behr (2020) [26]		1989–2018	ELSO	ELSO	Retrospective	Burns	121	NR	NR	NR	NR

*TBSA* total body surface area, *ELSO* extracorporeal life support organization, *NR* not reported

**Table 2:** Indications and outcomes of ECMO in included studies

**No.**	**Population**	**First author (year)**	**ECMO indication (*n*)**	**ECMO starting time**	**Mean ECMO duration**	**PaO**_**2**_ **/FiO**_**2**_ **before ECMO (mmHg)**	**ECMO mode (*n*)**	**Successful weaning (*n*)**	**Mortality (*n*)**	**Complications (*n*)**	**CRRT (*n*)**
1	Adult	Li (2021) [29]	Severe ARDS	10.2 d	180.4 h	111.8	VV ECMO (5)	3	4	Bleeding (4)	4
2	Adult	Szentgyorgyi (2018) [33]	Severe ARDS	7.4 d	18 d	67.6	VV ECMO (5)	5	1	Infection (1)	4
3	Adult	Dadras (2019) [32]	Severe ARDS	9 d	388 ± 283 h	62	VV ECMO (7), mixed (1)	5	3	Bleeding (1) limb ischemia (1), sepsis (3)	4
4	Adult	Ainsworth (2018) [36]	Severe ARDS	NR	276 h	82	VV ECMO (14)	10	6	Bleeding (10)	9
5	Adult	Hsu (2017) [37]	Severe ARDS (5), cardiac shock (1)	26.5 h	169.6 h	66.7	VV ECMO (2), VA ECMO (4)	1	5	NR	1
6	Adult	Chiu (2017) [35]	Severe ARDS	25 d	119 h	87.1	VV ECMO (4), mixed (1)	3	2	Limb ischemia (1), ECMO wound dehiscence (1)	4
7	Adult	Soussi (2016) [39]	Severe ARDS	4 d^a^	90 h	66	VV ECMO (8), VA ECMO (2), mixed (1)	NR	8	Bleeding (1)	NR
8	Adult	Marcus (2019) [30]	ARDS	NR	249 h	NR	VV ECMO (20)	14	8	Infection (20)	NR
9	Adult	Nehra (2009) [28]	NR	NR	NR	NR	NR	NR	7	NR	NR
10	Adult	Burke (2017) [38]	Severe ARDS	5 d^a^	185 h	NR	VV ECMO (44), VA ECMO (14)	NR	33	Bleeding (16), infection (12), renal failure (20)	32
11	Adult	Nosanov (2017) [34]	RF (9), ARDS (3)	NR	NR	NR	VV (11), VA (17), mixed (8)	NR	16	Sepsis (6), pneumonia (9)	6
12	Pediatrics	Eldredge (2019) [31]	Severe ARDS	7.5 d	11 d	59	VV ECMO (8)	NR	1	Bleeding (5)	NR
13	Pediatrics	Kane (1999) [27]	Severe ARDS	7.8 d	165.2 h	75.2	NR	NR	4	NR	NR
14	Pediatrics	Pierre (1998) [40]	ARDS	NR	324.4 h	NR	NR	NR	2	Bleeding (2), blood infection (1)	3
15	Pediatrics	Behr (2020) [26]	NR	NR	NR	NR	NR	NR	63	NR	NR

*d* days, *ECMO* extracorporeal membrane oxygenation, *ARDS* acute respiratory distress syndrome, *PaO_2_* arterial oxygen partial pressure, *FiO2* fraction of inspired oxygen, *RF* respiratory failure, *VV ECMO* veno–venous ECMO, *VA ECMO* veno–arterial ECMO, *CRRT* continuous renal replacement therapy, *NR* not reported

^a^Days after admission

### Primary outcome: in-hospital mortality

#### Pooled in-hospital mortality and subgroup analysis

**Figure 2:**
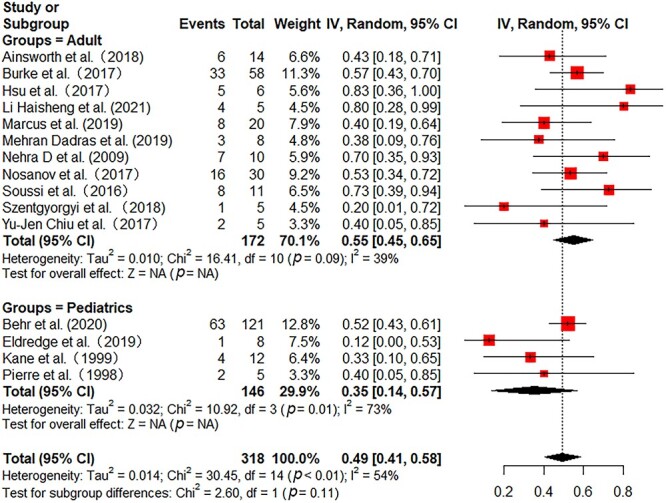
Forest plot of mortality on ECMO for adult and pediatric burn patients using a random-effect model. *ECMO* extracorporeal membrane oxygenation, *CI* confidence interval

**Figure 3:**
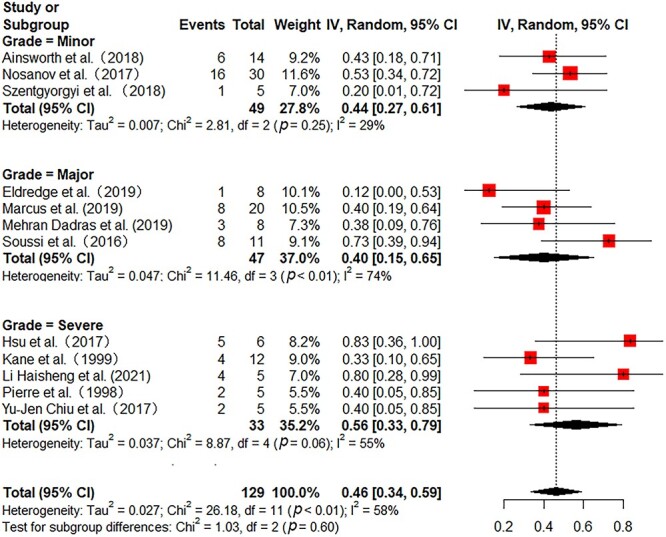
Forest plot of mortality on ECMO for burn patients with different burn severity using a random-effect model. Burn severity was defined as follows: minor burns: adult TBSA <30%, pediatric TBSA <15%; major burns: adult TBSA 30–50%, pediatric TBSA 15–30%; severe burns: adult TBSA >50%, pediatric TBSA >30%. *ECMO* extracorporeal membrane oxygenation, *CI* confidence interval, *TBSA* total body surface area

**Figure 4:**
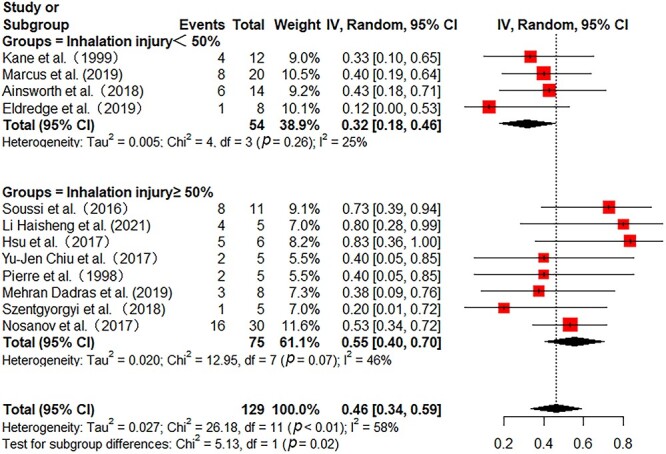
Forest plot of mortality on ECMO for burn patients with different percentage of inhalation injury using a random-effect model. Subgroup was stratified by the percentage of inhalation injury in different studies (≥50 *vs* <50%). *ECMO* extracorporeal membrane oxygenation, *CI* confidence interval

**Figure 5:**
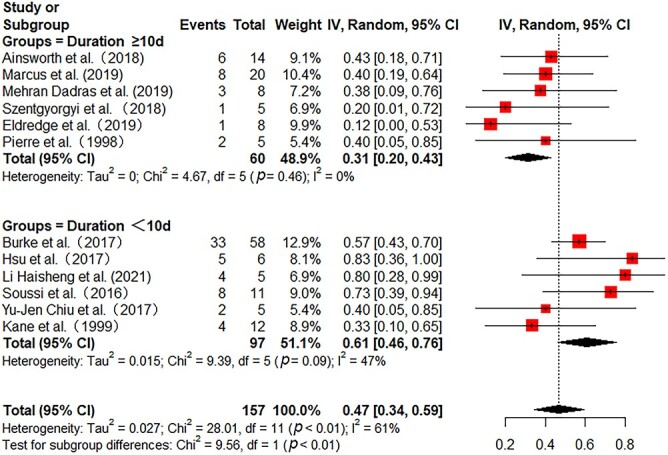
Forest plot of mortality on ECMO for burn patients with different ECMO duration. Subgroup was stratified by the ECMO duration in different studies (≥10 *vs* <10 days). *ECMO* extracorporeal membrane oxygenation, *CI* confidence interval

All the included studies reported in-hospital mortality. Pooled in-hospital mortality was 49% (95% CI 41–58%) with moderate heterogeneity among the studies (*I^2^* = 54%) (Figure 2). Sensitivity analyses found no single study greatly influenced the pooled results (supplementary Figure S1, see online supplementary material). Subgroup analyses were further performed to reduce heterogeneity. In 172 adult patients from 11 studies, the pooled mortality was 55% (95% CI 45–65%) with low heterogeneity (*I^2^* = 39%) (Figure 2). In 146 pediatric patients from 4 studies, the pooled mortality was 35% (95% CI 14–57%) with substantial heterogeneity (*I^2^* = 73%) (Figure 2). Then, we further analyzed the mortality in different burn areas. The pooled mortality in minor and major burn populations were respectively 44% (95% CI 27–61%, *I^2^* = 29%) and 40% (95% CI 15–65%, *I^2^* = 74%), which was lower than that in severe burn populations (56%, 95% CI 33–79%, *I^2^* = 55%) (Figure 3). As stratified by inhalation injury, the pooled mortality in studies with percentage of inhalation injury ≥50% (55%, 95% CI 40–70%, *I^2^* = 46%) was higher than that in studies with percentage of inhalation injury <50% (32%, 95% CI 18–46%, *I^2^* = 25%) (Figure 4). As for ECMO duration, the pooled mortality in studies with ECMO duration ≥10 days (31%, 95% CI 20–43%, *I^2^* = 0%) was lower than that in studies with ECMO duration <10 days (61%, 95% CI 46–76%, *I^2^* = 47%) (Figure 5).

#### Meta-regression analysis

**Figure 6:**
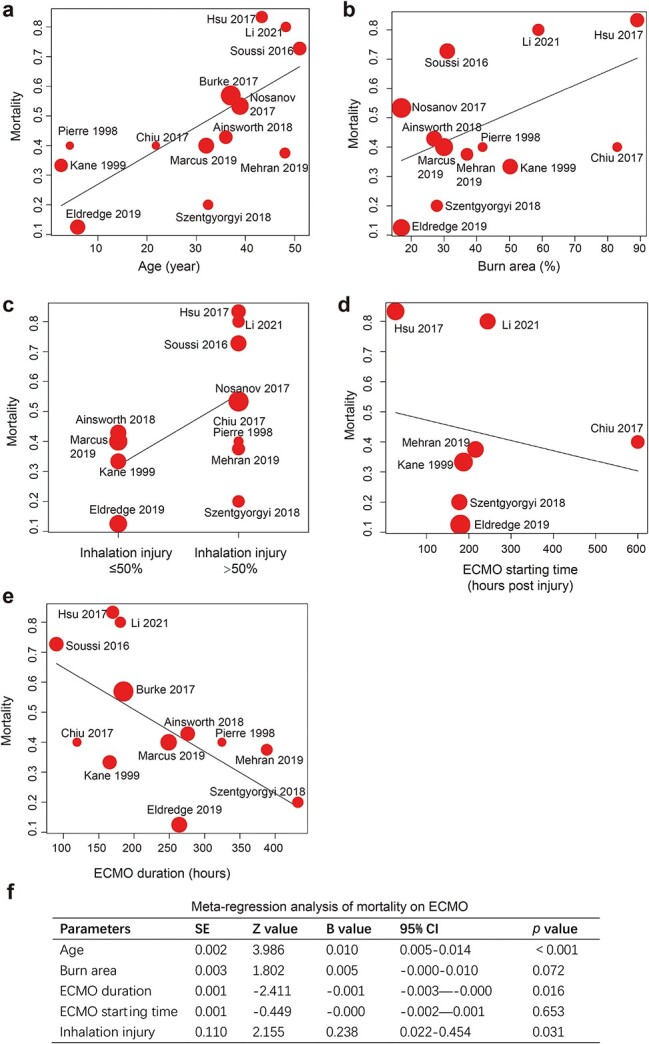
Bubble plot and meta-regression analysis of mortality on ECMO. (**a**) Bubble plot correlating age and mortality. (**b**) Bubble plot correlating burn area and mortality. (**c**) Bubble plot correlating percentage of inhalation injury and mortality. (**d**) Bubble plot correlating ECMO starting time and mortality. (**e**) Bubble plot correlating ECMO duration and mortality. (**f**) Summary result of meta-regression analysis of mortality. *ECMO* extracorporeal membrane oxygenation, *CI* confidence interval, *SE* standard error

Further meta-regression analysis showed that age (B value 0.010, *p* < 0.001), inhalation injury (B value 0.238, *p* = 0.031) and duration of ECMO (B value −0.001, *p* = 0.016) were the main contributors to the observed heterogeneity. Considering the high *I^2^* and great difference of sample sizes in the pediatric groups (Figure 2), age might not be the source of heterogeneity. Bubble plots and meta-regression analysis also supported that older age, larger burn area (B value 0.005, *p* = 0.072) and higher percentage of inhalation injury were associated with higher mortality (Figure 6). Later ECMO starting time (B value −0.000, *p* = 0.653) and longer ECMO duration were associated with lower mortality (Figure 6). Further interactive subgroup analysis revealed that studies with percentage of inhalation injury ≥50% and ECMO duration <10 days had the highest mortality and lowest heterogeneity (supplementary Figure S2, see online supplementary material).

### Secondary outcomes

#### Successful weaning off

**Figure 7:**
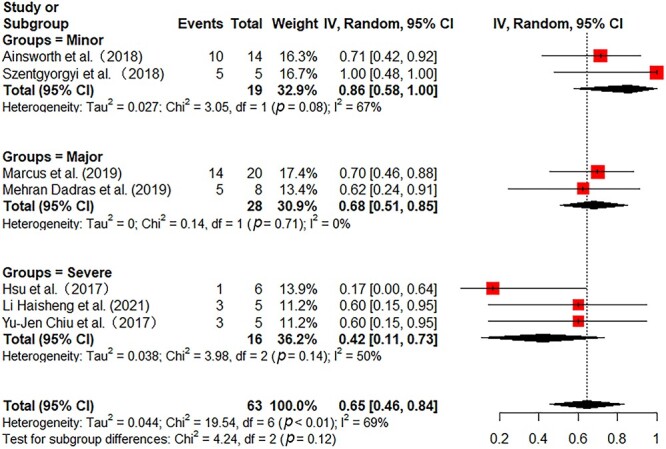
Forest plot of successful weaning from ECMO for burn patients with different severity using a random-effect model. *ECMO* extracorporeal membrane oxygenation, *CI* confidence interval

A total of 7 studies with 63 patients reported the percentage of successful weaning from ECMO [29,30,32,33,35–37] and the pooled percentage was 65% (95% CI 46–84%) with moderate heterogeneity (*I^2^* = 69%). Meta-regression analysis found that increasing burn area (B value −0.008, *p* = 0.004) was significantly associated with a decreasing rate of successful weaning from ECMO (Supplementary Figure S3, see online supplementary material). As shown in Figure 7, subgroup analysis of different burn severities showed that the percentage of successful weaning from ECMO decreased with an increase of burn area. Specifically, the pooled percentages of successful weaning from ECMO in minor, major and severe burn populations were 86 (95% CI 58–100%, *I^2^* = 67%), 68 (95% CI 51–85%, *I^2^* = 0%) and 42% (95% CI 11–73%, *I^2^* = 50%), respectively.

#### Complications

Complications during ECMO were presented in 11 studies (169 patients) [29–36,38–40]. There were 114 reported complications with a rate of 67.46%. Infectious complications (52/169) were the most common, followed by bleeding (39/169) and renal complications (20/169). A summary of all the ECMO-related complications is provided in Table 2. In all, 49.26% of patients (67/136) required continuous renal replacement therapy in 9 reported studies [29,32–38,40].

### Quality assessment

All the included studies had a JBI score >7 (Supplementary Table S2, see online supplementary material), revealing a high level of quality. Inadequate sample size and objective subpopulation analysis were the most common drawbacks. Funnel plots did not show obvious publication bias for mortality and successful weaning off (supplementary Figures S4 and S5, see online supplementary material). Egger’s tests also showed no significant evidence of publication bias (Supplementary Table S3, see online supplementary material). Supplementary Table S4 (see online supplementary material) summarizes GRADE’s determination of certainty of evidence.

## Discussion

The application of ECMO in the treatment of severe burns complicated by cardiopulmonary dysfunction has been increasing in recent years. However, the sample size of most studies was low and no consensus has been achieved on the initiation and management of ECMO in burn patients. Although a systematic review of ECMO in burn populations has previously been published, the researchers only briefly analyzed the clinical outcomes of mortality compared with the predicted mortality by the revised Baux score based on limited studies and sample sizes (82 patients in 14 articles) [17]. They did not investigate the safety and other clinical outcomes of ECMO. To our knowledge, this is the first comprehensive systematic and meta-analysis of clinical studies to investigate both the efficacy and the safety of ECMO in burn patients. Here, we found that the pooled mortality was 49% in burn patients, 55% in adult patients and 35% in pediatric patients. However, the impact of ECMO on mortality could not be defined because of the lack of a control group in all studies. Further subgroup analysis and meta-regression analysis also showed that mortality increased with age, burn area and inhalation injury, and decreased with starting time and duration of ECMO. The pooled percentage of successful weaning off was 65% and inversely correlated with burn area. The rate of ECMO-related complications was high (67.46%) in burn populations, and infection and bleeding were the two most common complications. Our results could provide valuable evidence for the application of ECMO in burn patients.

It seems tricky to define whether ECMO could decrease the mortality of burn patients because no control group was applied in all the reported studies. Chiu *et al*. [17] compared the mortality of patients receiving ECMO with the mortality predicted by the revised Baux score, and found that only burn patients with inhalation injuries and revised Baux scores >90 had a lower mortality than that predicted. The revised Baux score, along with the Abbreviated Burn Severity Index and the Prognostic Burn Index are common models for predicting burn mortality. However, they do not consider complications and comorbidities, and may have different prediction accuracies in different burn populations [41]. Furthermore, burn patients requiring ECMO usually have severe complications, such as organ dysfunction. Therefore, comparing the actual and predicted mortality has little clinical relevance. Another strategy is to refer to the reported mortality of burn patients with moderate or severe ARDS, because this study confirmed severe ARDS was the most common indication of ECMO in burn patients. For total intensive care unit patients with ARDS, the mortality of moderate ARDS and severe ARDS was 40.3% and 46.1%, respectively [42]. A recent systematic review found that the overall mortality of burn patients with ARDS was 27% [5]. As for the different subgroups of ARDS, the reported mortality of moderate ARDS was 36.1% [43] and 20.9% [44], and the mortality of severe ARDS was 43.8% [43] and 50% [44] in different studies. This study found that pooled hospital mortality was 49% in burn patients with ECMO. Therefore, we concluded that the efficacy of ECMO on the mortality of burns with ARDS might be little. Randomized control trial (RCT) studies are needed to clarify this question.

The pooled percentage of successful weaning off was higher than that of overall mortality. In clinical practice, successful weaning off ECMO usually means that the main purpose of ECMO, gas exchange or circulatory support, has been achieved. The difference between the percentage of successful weaning off and mortality meant some patients ultimately died from other causes, even if ARDS had been alleviated. This hinted that severe burn care needs to focus not only on one organ, but also needs to balance the whole condition. There might be some other factors influencing the efficacy of ECMO. Further subgroup analysis showed that pediatric burns and non-severe burns, without inhalation injury, had a lower mortality than adult burns and severe burns, with inhalation injury. Compared to patients with a duration of ECMO <10 days, patients with duration of ECMO >10 days had lower mortality, partly implying that most deaths occurred in the early stage of ECMO utilization. Meta-regression analysis also supported these findings. Meanwhile, age, TBSA and inhalation injury were listed as items of many common burn severity score systems [45,46]. Therefore, the efficacy of ECMO was closely associated with burn severity.

Apart from respiratory support, cardiac and circulatory support is another common application for ECMO, using VA ECMO mode. Acute cardiogenic shock caused by myocardiac diseases or surgery is the recommended indication for VA ECMO [47]. However, the death rate for VA ECMO was higher than for VV ECMO. In this study, only 37 patients in 4 included studies partly received VA ECMO [37–39,48]. Burke *et al.* found that the mortality in the VA ECMO group (11/14, 78.6%) was significantly higher than that in the VV ECMO group (22/44, 50%) [38]. In a study by Hsu *et al*. [37], all the 4 patients with VA ECMO died, compared to 1 of 2 patients with VV ECMO surviving. Other two studies did not report the details of patients with VA ECMO. This finding was also supported by other studies. In Extracorporeal Life Support Organization (ELSO)-registered centers, the reported in-hospital mortality rate for all adult ECMO patients was 51%, with respiratory and cardiac failure causing 42 and 55% of deaths respectively [49]. A recent systematic review found that VA ECMO in cardiogenic shock was associated with an in-hospital mortality rate of 62% [50]. Based on these findings, we concluded that the clinical efficacy of ECMO in circulatory support might be inferior to that in respiratory support.

Acute kidney injury is a frequent complication of ECMO. The incidence of acute kidney injury is as high as 70–85% in patients undergoing ECMO and is a main risk factor of death [51]. Therefore, CRRT is usually combined with ECMO for renal function replacement and also fluid management. During ECMO, the percentage of CRRT in burn patients was significantly higher than that in other ECMO patients (49.26 *vs* 20%) [52]. Fluid was administered to recover normovolemia, provide antibiotic therapy, maintain normal hemodynamics and treat bleeding complications. Fluid overload is common during ECMO and increases the load of renal function. A multicenter international survey showed that the indications for starting CRRT on ECMO were fluid overload treatment (43%), fluid overload prevention (16%), acute kidney injury (35%) and electrolyte imbalance (4%) [52,53]. However, the detailed indications, timing, anticoagulation strategy, infection, nutrition support and wound management varied between studies and need further optimization for burn patients [54].

ECMO-related complications were another important consideration. This study found that infection and bleeding were the major complications, which was consistent with other populations [55]. However, the occurrence rate of complications was higher in burn patients than in other populations (67.46 *vs* 40.2%) [55]. The pooled infection rate was 30.8%, within the range of 8 to 64% in adult populations with ECMO [56]. Moreover, one study found that the overall infection rate of burn patients was nearly 3-fold that of non-burn patients during ECMO [30], mainly attributed to burn wounds and destroyed skin barrier. Similar to other adult populations, respiratory infection was the main infection type during ECMO in burn patients, followed by bloodstream infection and wound infection [30,56]. Bleeding was another secondary common complication, with a rate of 23.1%. The underlying causes of hemorrhage include heparin application, deficiency of coagulation factors, coagulopathy, reduced number and function of platelets, and hyperfibrinolysis. Heparin-induced thrombocytopenia can also be caused by continuous infusion of heparin [57]. Wounds were the most common bleeding site, followed by cannulation site, craniocerebral, lung and gastrointestinal tract [36,38]. Local anticoagulants, regular monitoring and targeted management under the direction of an algorithm may be beneficial to prevent common bleeding [58]. Furthermore, another un-neglected compication was thrombosis which could be formed in the cannula, ECMO machine and deep veins [59]. Long duration of ECMO was the consistent risk factor for ECMO-related complications [55,59].

Some limitations exist in this study. First, all the included studies were retrospective and had no control group. Therefore, we could not clarify whether ECMO improved clinical outcomes or not. Moreover, there are inherent limitations to retrospective studies, such as selection bias and missing data. However, this study could at least provide important references for the safety and efficacy of ECMO in burn patients. Strict RCT studies are needed to get strong evidence. Second, case reports or case series with a sample size <5 were not included in this study, because such studies may have strong publication and selection bias. As shown in supplementary Table S5 (see online supplementary material), only 3 out of 37 patients died in the case reports, which was much lower than in cohort studies. Some cases might also be included in other studies.

## Conclusions

In conclusion, this study supports that ECMO is a suitable rescue therapy for burn patients despite the slightly high mortality (49%). Increasing age and burn area, and inhalation injury were risk factors for death. Prolonged duration of ECMO and late starting time appeared to be protective factors against death. The occurrence rate of ECMO-related complications was also high in burn populations. Infection and bleeding were the two most common complications. More RCT studies are needed to verify the efficacy and safety of ECMO in burn patients in the future.

**Abbreviations**
ARDS: Acute respiratory distress syndrome; CIs: Confidence intervals; CRRT: Continuous renal replacement therapy; ECMO:
  Extracorporeal membrane oxygenation; ELSO: Extracorporeal Life Support Organization; FiO_2_: Fraction of inspired oxygen; GRADE: Grading of Recommendations, Assessments, Developments and Evaluations. JBI: Joanna Briggs Institute; PaO_2_: Arterial oxygen partial pressure; PRISMA: Preferred Reporting Items for Systematic Reviews and Meta-Analysis; RCTs: Randomized controlled trials; VA ECMO:
  Veno–arterial ECMO; VV ECMO: Veno–venous ECMO.

## Supplementary Material

Supplementary_tables_and_figuresR2_tkac056

PRISMA_2020_checklist_tkac056

## Data Availability

All generated or analyzed data of the present study are included in this article and its supplementary materials.

## Coonflicts of interest

None declared.
