# Supplementary material for: Efficacy and safety of extracorporeal membrane oxygenation for burn patients: a comprehensive systematic review and meta-analysis
Source: Burns Trauma. 2023 Mar 1;11:tkac056. doi: 10.1093/burnst/tkac056 (PMC9977350; doi:10.1093/burnst/tkac056)
Supplement: Supplementary_tables_and_figuresR2_tkac056 [file supplementary_tables_and_figuresr2_tkac056.doc]

**1. Supplementary figures**


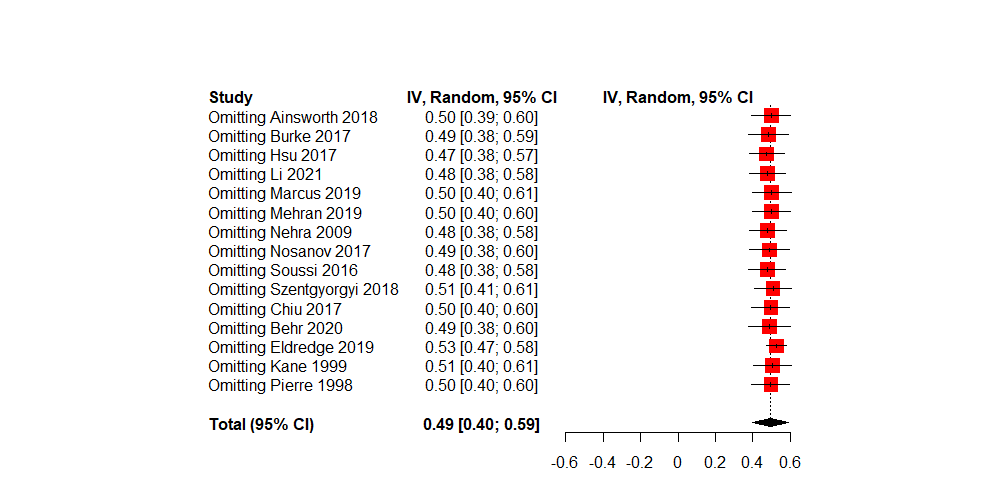


**Figure S1. Sensitivity analyses of studies reporting on ECMO mortality**

*ECMO* extracorporeal membrane oxygenation, *CI* confidence interval

**
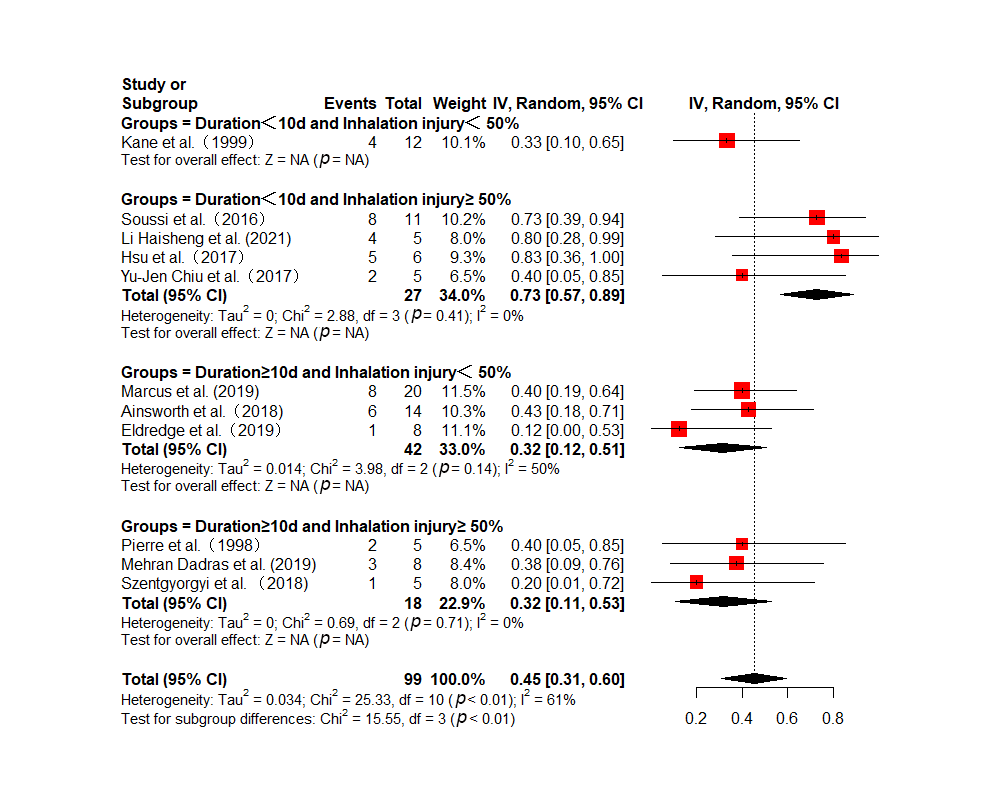
**

**Figure S2. Interactive subgroup analysis of mortality on ECMO by inhalation injury and ECMO duration.** *ECMO* extracorporeal membrane oxygenation, *CI* confidence interval


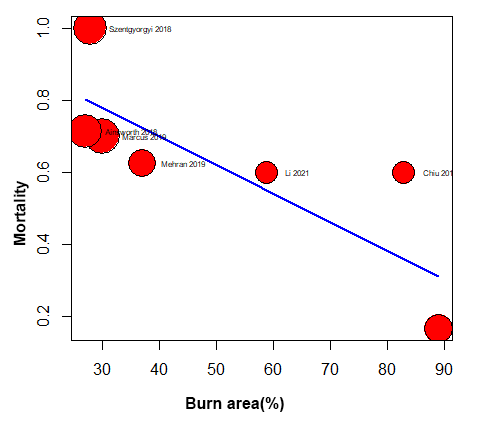


**Figure S3. Bubble plot correlating rate of successful weaning from ECMO with burn area.** *ECMO* extracorporeal membrane oxygenation

**
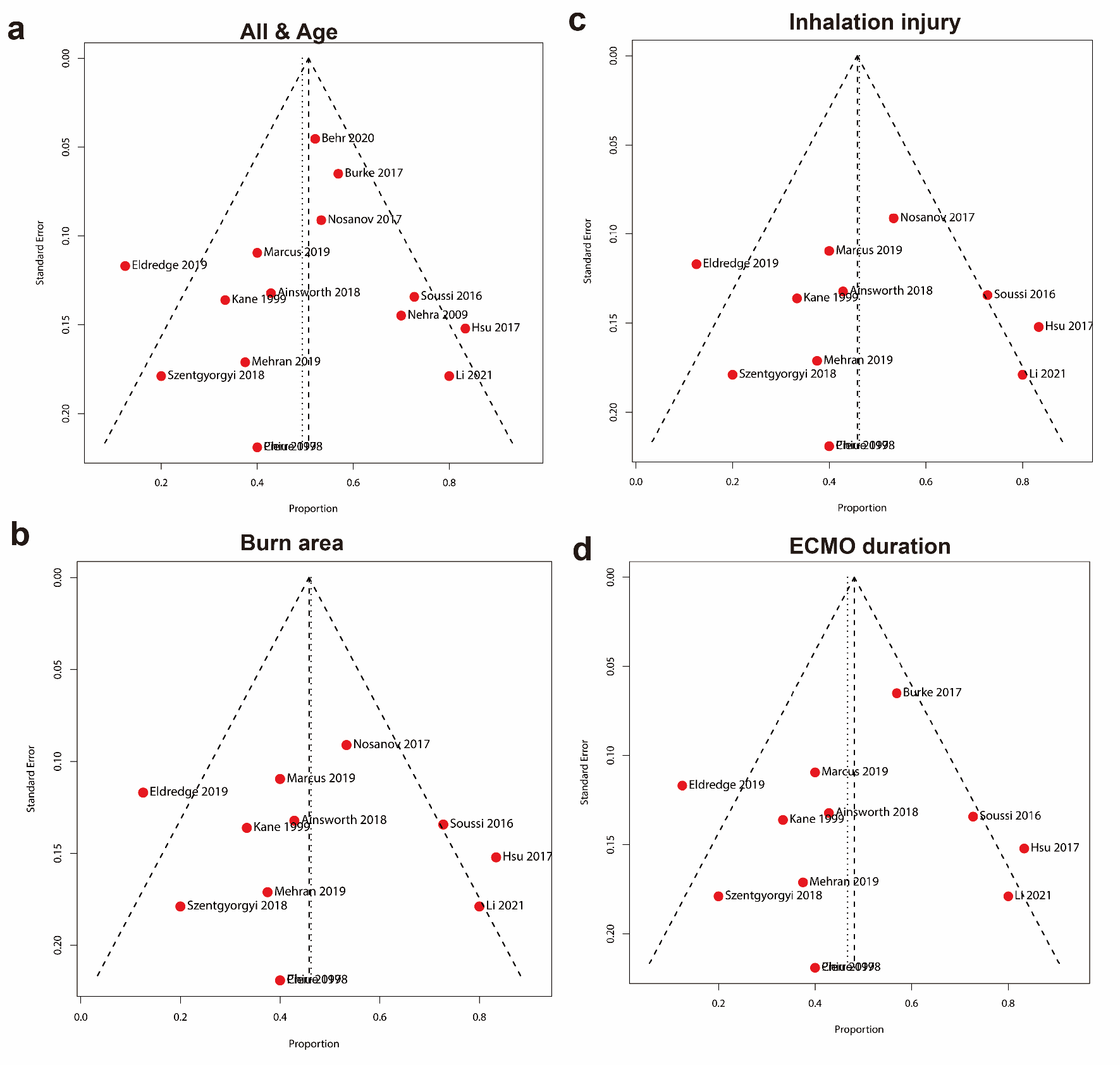
**

**Figure S4. Funnel plot of studies reporting on ECMO mortality**

(a) Funnel plot of all included studies. (b). Funnel plot of subgroup analysis stratified by burn area. (c) Funnel plot of subgroup analysis stratified by percentage of inhalation injury. (d) Funnel plot of subgroup analysis stratified by ECMO duration. *ECMO* extracorporeal membrane oxygenation


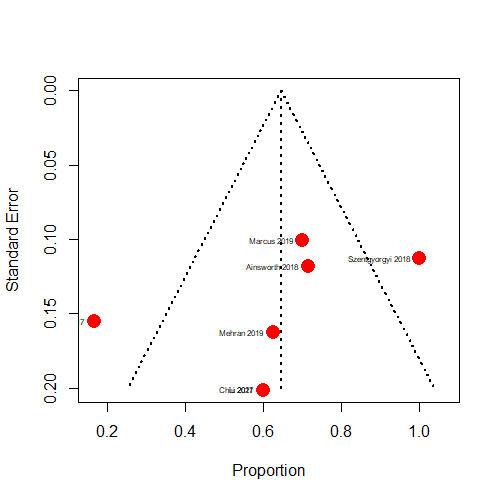


**Figure S5. Funnel plot of studies reporting on rate of successful weaning from ECMO**. *ECMO* extracorporeal membrane oxygenation

**2. Supplementary tables**

**Table S1**. Search strategy

| **Database: PubMed**  Data searched: 18 March 2022  Records retrieved: **96**  **#Burn**  "burns"[Title/Abstract] OR "burn"[Title/Abstract] OR "inhalation injury"[Title/Abstract]  **#ECMO**  (ECMO*[Title/Abstract]) OR "extracorporeal membrane oxygenation"[Title/Abstract] OR "extra corporeal membrane oxygenation"[Title/Abstract] OR "extra-corporeal membrane oxygenation"[Title/Abstract] OR ECLS*[Title/Abstract] OR “extracorporeal oxygenation”[Title/Abstract] OR “extra-corporeal oxygenation”[Title/Abstract] OR “extra corporeal oxygenation”[Title/Abstract] OR "extracorporeal life support"[Title/Abstract] OR "extra corporeal life support"[Title/Abstract] OR "extra-corporeal life support"[Title/Abstract])  **#Total**  **#Burn AND #ECMO**  ("ECMO"[Title/Abstract] OR "extracorporeal membrane oxygenation"[Title/Abstract] OR "extra corporeal membrane oxygenation"[Title/Abstract] OR "extra corporeal membrane oxygenation"[Title/Abstract] OR "ECLS"[Title/Abstract] OR "extracorporeal oxygenation"[Title/Abstract] OR "extra corporeal oxygenation"[Title/Abstract] OR "extra corporeal oxygenation"[Title/Abstract] OR "extracorporeal life support"[Title/Abstract] OR "extra corporeal life support"[Title/Abstract] OR "extra corporeal life support"[Title/Abstract] OR "extracorporeal membrane oxygenation"[Title/Abstract]) **AND** ("burns"[Title/Abstract] OR "burn"[Title/Abstract] OR "inhalation injury"[Title/Abstract]) |
| --- |
| **Database: Embase**  Data searched: 18 March 2022  Records retrieved: 436   | No. | Query | | --- | --- | | #1 | 'extracorporeal oxygenation'/exp | | #2 | 'extracorporeal oxygenation' | | #3 | 'extracorporeal membrane oxygenation' | | #4 | 'extracorporeal' AND 'membrane' AND 'oxygenation' | | #5 | ecmo | | #6 | #1 OR #2 OR #3 OR #4 OR #5 | | #7 | 'burn'/exp | | #8 | 'burn' | | #9 | 'burns' | | #10 | 'inhalation injury' | | #11 | #7 OR #8 OR #9 OR #10 | | #12 | #6 AND #11 | |
| **Database: Web of science**  Data searched: 18 March 2022  Records retrieved: 340  **#1**  **((TS=(burns)) OR TS=(burn)) OR TS=(inhalation injury)**  **#2**  **(((((((((**(TS=(ECMO)) OR TS=(extracorporeal membrane oxygenation)) OR TS=(extra corporeal membrane oxygenation)) OR TS=(extra-corporeal membrane oxygenation)) OR TS=(ECLS)) OR TS=(extracorporeal oxygenation)) OR TS=(extra-corporeal oxygenation)) OR TS=(extra corporeal oxygenation)) OR TS=(extracorporeal life support)) OR TS=(extra corporeal life support)) OR TS=(extra-corporeal life support)  **#3**  **#1 AND #2** |

**Table S2 Quality assessment of included studies by JBI scores**

| **Studies** | **Was the sample representative of the target population?** | **Were study participants recruited in an appropriate way?** | **Was the sample size adequate?** | **Were the study subjects and setting described in detail?** | **Is the data analysis conducted with sufficient coverage of the identified sample?** | **Were objective, standard criteria used for measurement of the condition?** | **Was the condition measured reliably?** | **Was there appropriate statistical analysis?** | **Are all important confounding factors/ subgroups/differences identified and accounted for?** | **Were subpopulations identified using objective criteria?** | **Total score** |
| --- | --- | --- | --- | --- | --- | --- | --- | --- | --- | --- | --- |
| Ainsworth（2018） | 1 | 1 | 1 | 1 | 1 | 1 | 1 | 1 | 1 | 1 | 10 |
| Burke（2017） | 1 | 1 | 1 | 1 | 1 | 1 | 1 | 1 | 1 | 1 | 10 |
| Hsu（2017） | 1 | 1 | 0 | 1 | 1 | 1 | 1 | 1 | 1 | 1 | 9 |
| Li Haisheng(2021) | 1 | 1 | 0 | 1 | 1 | 1 | 1 | 1 | 1 | 1 | 9 |
| Marcus(2019) | 1 | 1 | 1 | 1 | 1 | 1 | 1 | 1 | 1 | 1 | 10 |
| Mehran Dadras(2019) | 1 | 1 | 0 | 1 | 1 | 1 | 1 | 1 | 1 | 0 | 8 |
| Nehra D(2009) | 1 | 1 | 1 | 1 | 1 | 1 | 1 | 1 | 1 | 1 | 10 |
| Nosanov （2017） | 1 | 1 | 1 | 1 | 1 | 1 | 1 | 1 | 1 | 1 | 10 |
| Soussi（2016） | 1 | 1 | 1 | 1 | 1 | 0 | 1 | 1 | 1 | 0 | 8 |
| Szentgyorgyi (2018) | 1 | 1 | 0 | 1 | 1 | 1 | 1 | 1 | 1 | 0 | 8 |
| Chiu(2017) | 1 | 1 | 0 | 1 | 1 | 1 | 1 | 1 | 1 | 1 | 9 |
| Behr (2020) | 1 | 1 | 1 | 1 | 1 | 1 | 1 | 1 | 1 | 1 | 10 |
| Eldredge（2019） | 1 | 1 | 0 | 1 | 1 | 1 | 1 | 1 | 1 | 1 | 9 |
| Kane （1999） | 1 | 1 | 1 | 1 | 1 | 1 | 1 | 1 | 1 | 0 | 9 |
| Pierre (1998) | 1 | 1 | 0 | 1 | 1 | 1 | 1 | 1 | 1 | 0 | 8 |
| *JBI* Joanna Briggs Institute | | | | | | | | | | | |

**Table S3 Egger’s test results for publication bias**

| **Groups** | **p-value** |
| --- | --- |
| **Mortality-all studies** | 0.6513 |
| **Mortality-burn severity** | 0.8708 |
| **Mortality-inhalation injury** | 0.8708 |
| **Mortality-ECMO duration** | 0.6607 |
| **Successful weaning off** | 0.2709 |

*ECMO* extracorporeal membrane oxygenation

**Table S4. GRADE assessment of results of meta-analysis for use of ECMO in burn patients**

| **No of studies** | **Certainty assessment** | | | | | | |  | **Effects** | | | **Certainty** | **Importance** |
| --- | --- | --- | --- | --- | --- | --- | --- | --- | --- | --- | --- | --- | --- |
|  | **Study design** | **Risk of bias** | **Inconsistency** | **Indirectness** | **Imprecision** | **Publication bias** | **Other considerations** |  | **No. of events** | **No. of individuals** | **Rate (95% CI)** |  |  |
| ECMO mortality  15 | Observational studies | Seriousa | Not seriousb | Not serious | Not serious | Undetected | None |  | 163 | 318 | 0.49 (0.40-0.59) | ⊕⊕⊕◯  Moderate | Critical |
| Rate of Successful  Weaning off  7 | Observational studies | Seriousa | Not serious^c^ | Not serious | Not serious | Undetected | None |  | 41 | 63 | 0.65 (0.45-0.84) | ⊕⊕⊕◯  Moderate | Critical |
| a There was a lack of a control (non-exposed) group in all studies and a low level of comparability in the majority of the included studies  b There was some heterogeneity (I^2^ = 54%) in the point estimates. Nonetheless, the 95% CIs for the individual studies mostly overlapped with each other.  c There was substantial heterogeneity (I^2^ = 69%) in the point estimates. Nonetheless, the 95% CIs for the individual studies mostly overlapped with each other.  *ECMO* extracorporeal membrane oxygenation, *CI* confidence interval | | | | | | | | | | | | | |

**Table S5 Overview of case report and case series (sample size<5)**

| **Population** | **First author (Year)** | **Sample size** | **Gender** | **Median age (years)** | **Median burn area (TBSA）** | **Inhalation injury (n)** | **ECMO indication** | **ECMO starting time** | **ECMO duration** | **ECMO mode** | **Successful weaning off (n)** | **Mortality, n(%)** | **Complications** | **CRRT (n)** |
| --- | --- | --- | --- | --- | --- | --- | --- | --- | --- | --- | --- | --- | --- | --- |
| Adult | [Dun Yu](https://www.ncbi.nlm.nih.gov/pubmed/?term=Yu D%5BAuthor%5D&cauthor=true&cauthor_uid=34660650)(2021)[1] | 2 | Male | 43 | NR | 2 | ARDS | NR | 5d | VV ECMO | 2 | 0 | NR | NR |
| Adult | Piper LC(2021)[2] | 1 | Male | 34 | NR | NR | Severe hypoxemia | NR | 4d | VV ECMO | 1 | 0 | NR | NR |
| Adult | Ji Hoon Jang(2021)[3] | 1 | Male | 34 | NR | 1 | Hypoxemia | 1d | 8d | VV ECMO | 1 | 0 | NR | 1 |
| Adult | Li Y(2020)[4] | 1 | Male | 44 | NR | 1 | Severe ARDS | 17h | 6d | VV ECMO | 1 | 0 | NR | 1 |
| Adult | Jack Rasmussen(2020)[5] | 1 | Male | 38 | 80 | 1 | Severe ARDS | 4d | 14d | VV ECMO | 1 | 0 | Metabolic acidosis, renal injury | 1 |
| Adult | Derek C Lumbard (2020)[6] | 1 | Male | 47 | NR | NR | Cardiac arrest | 3h | 2d | VA ECMO | 1 | 0 | NR | 1 |
| Adult | Tamer Jamal（2020）[7] | 1 | Male | 26 | NR | NR | Cardiogenic shock | 30min | 86h | VA ECMO | 1 | 0 | NR | NR |
| Adult | Zhao Z(2019)[8] | 3 | Male | NR | NR | 3 | ARDS | NR | 19d | NR | 2 | 1(33.3) | NR | NR |
| Adult | Fang H (2019)[9] | 1 | Male | 24 | 60 | 1 | ARDS | 7h | 8d | NR | 1 | 0 | NR | NR |
| Adult | Lu H (2019)[10] | 1 | Male | 61 | NR | 1 | ARDS | 15h | 8d | VV ECMO | 1 | 0 | NR | NR |
| Adult | Davis CA(2018)[11] | 1 | Male | 58 | 1 | 1 | Severe hypoxemia | 1d | 8d | VV ECMO | 1 | 0 | NR | NR |
| Adult | Ray JJ(2018) [12] | 1 | Male | 27 | 60 | NR | ARDS. | 9d | 10d | VV ECMO | 1 | 0 | NR | NR |
| Adult | Pu Q(2017) [13] | 1 | Male | 25 | 60 | 1 | Cardiac insuffificiency, severe hypoxemia | 38h | 10d | VA ECMO | 1 | 0 | Infection | 1 |
| Adult | Kennedy(2017)[14] | 2 | Male | 46 | 32.5 | 0 | Hypoxemia. severe ARDS | 10d | 14d | VV ECMO | 2 | 0 | NR | NR |
| Adult | Kim Y-d(2017)[15] | 1 | Female | 36 | NR | 1 | Acute airway obstruction | NR | NR | NR | 1 | 0 | NR | NR |
| Adult | Lee YS（2012）[16] | 1 | Male | 42 | NR | 1 | ARDS | 4h | 7d | VV ECMO | 1 | 0 | Hypotension, tachycardia | NR |
| Adult | Thompson JT(2005)[21][17] | 2 | Male (1) female(1) | 33 | 17.5 | 1 | Severe ARDS | 4.5d | 294h | VV ECMO | 2 | 0 | Epistaxis, acute kidney injury, pneumonia | 1 |
| Adult | Chou NK (2001)[18] | 3 | Male (2) female (1) | 30.3 | 48.9 | 2 | ARDS Mocardial dysfunction | 16.2h | 160.2 h | VV ECMO (2), VV-VA ECMO (1) | 2 | 1(33.3) | Infection(3) | NR |
| Adult | Patton ML（1998）[19] | 1 | Male | 26 | 12 | 1 | Severe ARDS | NR | 80h | VV ECMO | 1 | 0 | Bleeding | NR |
| Pediatrics | Holton(2021)[20] | 1 | Female | 3 | 22 | NR | ARDS, shock | 7d | 87d | VV-VA ECMO | NR | NR | Bleeding, renal failure | 1 |
| Pediatrics | Harischandra T(2020)[21] | 1 | Female | 11 | NR | 1 | ARDS | 3d | 238h | VV ECMO | 1 | 0 | Infection | NR |
| Pediatrics | Kristen(2020)[22] | 1 | Female | 7 | 35 | 1 | Cardiac arrest | 7d | 2 years | VA-VV ECMO | 1 | 0 | Pneumonia, bleeding, renal failure, cardiac arrests, septic shock, clotting | 1 |
| Pediatrics | Panarello G (2015)[23] | 1 | Female | 1.4 | NR | 1 | Severe ARDS | 4d | 19 | VV ECMO | 1 | 0 | NR | NR |
| Pediatrics | Mangat HS (2012)[24] | 1 | Male | 2.6 | 45 | NR | Severe ARDS | 4d | 108h | NR | 0 | 1(100) | Bleeding | NR |
| Pediatrics | Cedidi C(2003)[25] | 1 | Male | 15 | 25 | 1 | Cardiac arrest, hypoxemia | 4d | 21d | VV ECMO | 1 | 0 | Pneumonia | 1 |
| Pediatrics | O'TooleG(1998) [26] | 2 | Male (1) female (1) | 1.6 | 10 | 2 | ARDS. | 8.5d | 4.5d | VV ECMO | 2 | 0 | NR | NR |
| Pediatrics | Lessin MS(1996)[27] | 2 | Male | 1.4 | 27.5 | 2 | Severe hypoxemia | 4d | 10d | VA ECMO | 2 | 0 | Bleeding (2) | NR |
| Pediatrics | Ombrellaro M (1994) [28] | 1 | Male | 0.9 | 32 | 1 | ARDS. | NR | 28d | NR | 1 | 0 | 0 | NR |

*ECMO* extracorporeal membrane oxygenation, *ARDS* acute respiratory distress syndrome, *VV ECMO* venovenous ECMO, *VA ECMO* veno-arterial ECMO, *CRRT* continuous renal replacement therapy, *NR* not reported

**References:**

[1] Yu D,Xiaolin Z,Lei P,Feng L,Lin Z,Jie S. Extracorporeal Membrane Oxygenation for Acute Toxic Inhalations: Case Reports and Literature Review. *Frontiers in medicine.* 2021; *8*: 745555. 10.3389/fmed.2021.745555.

[2] Piper L C,Nam J J,Kuckelman J P,Sams V G,DellaVolpe J D,Biscotti M,Negaard K A,Mason P E,Gurney J M. A Case Report of Combat Blast Injury Requiring Combat Casualty Care, Far-Forward ECMO, Air Transport, and All Levels of Military Critical Care. *Military medicine.* 2021, Aug 28:usab354. 10.1093/milmed/usab354.

[3] Jang JH,Jang HJ,Kim HK,Park JH,Kim HJ,Jo KM,Heo W,Kim SH,No T H,Lee JH. Acute respiratory distress syndrome caused by carbon monoxide poisoning and inhalation injury recovered after extracorporeal membrane oxygenation along with direct hemoperfusion with polymyxin B-immobilized fiber column: a case report. *Journal of medical case reports.* 2021; *15*: 456.

[4] Li Y,Cao C,Huang L,Xiong H,Mao H,Yin Q,Luo X. "Awake" Extracorporeal Membrane Oxygenation Combined With Continuous Renal Replacement Therapy For the Treatment of Severe Chemical Gas Inhalation Lung Injury. *Journal of burn care & research.* 2020; *41*: 908-912.

[5] Rasmussen J,Erdogan M,Loubani O,Green R S. Successful Use of Extracorporeal Membrane Oxygenation Therapy in Patients With 80% Full Thickness Burns. *Journal of burn care & research.* 2021; *42*: 345-347.

[6] Lumbard D C,Lacey A M,Endorf F W,Gayken J R,Fey R M,Schmitz K R,Deisler R F,Calcaterra D,Prekker M,Nygaard R M. Severe Hypothermia and Frostbite Requiring ECMO and Four Limb Amputations. *Journal of burn care & research.* 2020; *41*: 1301-1303.

[7] Jamal T,Shalabi A,Grosman-Rimon L,Ghanim D,Amir O,Kachel E. Veno-arterial extracorporeal membrane oxygenation for electrical injury induced cardiogenic shock support: a case report. *Journal of cardiothoracic surgery.* 2020; *15*: 143.

[8] Zhao Z, Xiaoyang Hong, et al. Extracorporeal membrane oxygenation: an efficient approach to treat acute respiratory distress syndrome after inhalation of zinc chloride from smoke bomb: a case report. *International Journal of Clinical and Experimental Medicine*. 2019;12:2042-7.

[9] Fang H,Wang G Y,Wang X,He F,Su J D. Potentially fatal electrolyte imbalance caused by severe hydrofluoric acid burns combined with inhalation injury: A case report. *World journal of clinical cases.* 2019; *7*: 3341-3346.

[10] Lu H,Liu Q,Lan C. Application of extracorporeal membrane oxygenation technique in patients with acute respiratory failure caused by ammonia poisoning. *Zhonghua wei zhong bing ji jiu yi xue.* 2019; *31*: 1542-1544.

[11] Davis C A,Paladino A D,Lassiter W B,Sharma A,Brady K. M. Intraoperative Venovenous Extracorporeal Membrane Oxygenation as Rescue for a Patient With an Inhalational Burn and Iatrogenic Upper Airway Injury: A Case Report. *A&A practice.* 2018; *11*: 115-117.

[12] Ray J J,Straker R J,Hart V J,Meizoso J P,Schulman C I,Loebe M,Ghodsizad A. The Use of Extracorporeal Membrane Oxygenation for Acute Respiratory Distress Syndrome in Severe Burns Without Inhalation Injury. *Journal of burn care & research.* 2018; *39*: 640-644.

[13] Pu Q,Qian J,Tao W,Yang A,Wu J,Wang Y. Extracorporeal membrane oxygenation combined with continuous renal replacement therapy in cutaneous burn and inhalation injury caused by hydrofluoric acid and nitric acid. *Medicine.* 2017; *96*: e8972.

[14] Kennedy J D,Thayer W,Beuno R,Kohorst K,Kumar A B. ECMO in major burn patients: feasibility and considerations when multiple modes of mechanical ventilation fail. *Burns & trauma.* 2017; *5*: 20. 10.1186/s41038-017-0085-9.

[15] Kim Y-d, Byunghoon Y, Cheol KM. Anesthetic management with extracorporeal membrane oxygenation in a patient with acute airway obstruction after inhalation burn injury -A case report. *Anesthesia and Pain Medicine* .2017, 12(3): 251-255.

[16] Lee Y S,Lee S H,Kim W Y,Kim J H,Park Y C. Anesthetic management of a patient with nitric acid inhalation injury for extracorporeal membrane oxygenation. *Korean journal of anesthesiology.* 2012; *62*: 194-195.

[17] Thompson J T,Molnar J A,Hines M H,Chang M C,Pranikoff T. Successful management of adult smoke inhalation with extracorporeal membrane oxygenation. *The Journal of burn care & rehabilitation.* 2005; *26*: 62-66.

[18] Chou N K,Chen Y S,Ko W J,Huang S C,Chao A,Jan G J,Lin F Y,Wang S S,Chu S H. Application of extracorporeal membrane oxygenation in adult burn patients. *Artificial organs.* 2001; *25*: 622-626.

[19] Patton M L,Simone M R,Kraut J D,Anderson H L, 3rd,Haith L R, Jr. Successful utilization of ECMO to treat an adult burn patient with ARDS. *Burns.* 1998; *24*: 566-568.

[20] Holton C,Orrick J,Areinamo I. Severe diastolic dysfunction following prolonged extracorporeal membrane oxygenation in and pediatric burn patient. *Chest* .*2021*; 160:A1942.

[21] Harischandra T,Withanaarachchi K,Piyasiri B,Wickramasuriya H,Piyasiri G,Firmin R. Successful use of extracorporeal membrane oxygenation in acute respiratory distress syndrome following accidental chlorine gas inhalation at a swimming pool. *Perfusion.* 2020; *35*: 543-545.

[22] Nelson-McMillan K,Vricella L A,Stewart F D,Young J,Shah A S,Hibino N,Coulson J D. Recovery from Total Acute Lung Failure After 20 Months of Extracorporeal Life Support. *ASAIO journal.* 2020; *66*: e11-e14.

[23] Panarello G,Occhipinti G,Piazza M,Capitanio G,Vitulo P,Gridelli B,Pilato M,Arcadipane A. Severe Acute Respiratory Failure due to Inhalation of Baby Powder and Successfully Treated with Venous-Venous Extracorporeal Membrane Oxygenation. *A & A case reports.* 2015; *5*: 228-230.

[24] Mangat H S,Stewart T L,Dibden L,Tredget E E. Complications of chlorine inhalation in a pediatric chemical burn patient: a case report. *Journal of burn care & research.* 2012; *33*: e216-221.

[25] Cedidi C,Hierner R,Pichlmaier M,Forssmann W G,Meyer M. Survival of severe ARDS with five-organ system failure following burns and inhalation injury in a 15-year-old patient. *Burns.* 2003; *29*: 389-394.

[26] O'Toole G,Peek G,Jaffe W,Ward D,Henderson H,Firmin R K. Extracorporeal membrane oxygenation in the treatment of inhalation injuries. *Burns.* 1998; *24*: 562-565.

[27] Lessin M S,el-Eid S E,Klein M D,Cullen M L. Extracorporeal membrane oxygenation in pediatric respiratory failure secondary to smoke inhalation injury. *Journal of pediatric surgery.* 1996; *31*: 1285-1287.

[28] Ombrellaro M,Goldthorn J F,Harnar T J,Shires G T. Extracorporeal life support for the treatment of adult respiratory distress syndrome after burn injury. *Surgery.* 1994; 115: 523-526.
